# Alpha-enolase on apical surface of renal tubular epithelial cells serves as a calcium oxalate crystal receptor

**DOI:** 10.1038/srep36103

**Published:** 2016-10-31

**Authors:** Kedsarin Fong-ngern, Visith Thongboonkerd

**Affiliations:** 1 Medical Proteomics Unit, Office for Research and Development, Faculty of Medicine Siriraj Hospital, and Center for Research in Complex Systems Science, Mahidol University, Bangkok, Thailand

## Abstract

To search for a strategy to prevent kidney stone formation/recurrence, this study addressed the role of α-enolase on apical membrane of renal tubular cells in mediating calcium oxalate monohydrate (COM) crystal adhesion. Its presence on apical membrane and in COM crystal-bound fraction was confirmed by Western blotting and immunofluorescence staining. Pretreating MDCK cells with anti-α-enolase antibody, not isotype-controlled IgG, dramatically reduced cell-crystal adhesion. Immunofluorescence staining also confirmed the direct binding of purified α-enolase to COM crystals at {121} > {100} > {010} crystal faces. Coating COM crystals with urinary proteins diminished the crystal binding capacity to cells and purified α-enolase. Moreover, α-enolase selectively bound to COM, not other crystals. Chemico-protein interactions analysis revealed that α-enolase interacted directly with Ca^2+^ and Mg^2+^. Incubating the cells with Mg^2+^ prior to cell-crystal adhesion assay significantly reduced crystal binding on the cell surface, whereas preincubation with EDTA, a divalent cation chelator, completely abolished Mg^2+^ effect, indicating that COM and Mg^2+^ competitively bind to α-enolase. Taken together, we successfully confirmed the role of α-enolase as a COM crystal receptor to mediate COM crystal adhesion at apical membrane of renal tubular cells. It may also serve as a target for stone prevention by blocking cell-crystal adhesion and stone nidus formation.

Due to the polarized characteristics of renal tubular epithelial cells, their apical membranes directly contact with tubular fluid and thus are involved with COM crystal adhesion, which is one of the initial mechanisms for kidney stone formation^1^,^2^. Recently, a number of potential COM crystal-binding molecules and/or proteins expressed on the apical membranes of renal tubular epithelial cells have been identified^2^,^3^,^4^. After renal tubular cell injury by numerous inducers, the injured renal tubular cells showed increased expression of COM crystal-binding molecules/proteins in concordance with the enhanced COM crystal binding on the cell surfaces^5^. Therefore, identification and characterizations of crystal-binding molecules/proteins on apical membranes of renal tubular epithelial cells may make kidney stone prevention feasible.

Our previous expression proteomics study successfully identified a large number of COM crystal-binding proteins isolated from apical membranes of MDCK renal tubular epithelial cells^4^. Among them, a glycolytic enzyme α-enolase was also identified by mass spectrometry in the COM crystal-bound fraction. Α-enolase is a 47-kDa enzyme that plays multiple roles in various cellular processes, including growth control, glycolysis and hypoxic tolerance^6^. Over the last few years, growing evidence has demonstrated that α-enolase is localized not only in cytoplasm but also on the cell surface of a variety of eukaryotic cells at which enzymatic catalytic activity remains^7^. Surface α-enolase also has a role in plasminogen-binding activity and serves as a plasminogen receptor, which is important for the development of some cancers^8^,^9^. This protein has increased expression level in the injured and regenerating cells during wound healing process^10^,^11^. In kidney stone disease, increasing evidence has pointed out its significance in kidney stone formation. High oxalate and testosterone treatments, both of which are the stone aggravators, increase expression level of α-enolase in renal tubular cells^12^,^13^, whereas epigallocatechin gallate (EGCG), a stone suppressor from both *in vitro* and *in vivo* studies, decreases α-enolase level in renal tubular cells^14^. Expression and additional data from these studies suggest that α-enolase may serve as a potential COM crystal receptor to mediate crystal binding on the cell surface. Nevertheless, the precise role of α-enolase as a receptor for COM crystals has not been confirmed. This study thus aimed to validate the role of α-enolase as a COM crystal receptor on apical membranes of renal tubular epithelial cells by using Western blotting, immunofluorescence staining, laser-scanning confocal microscopy, cell-crystal adhesion assay, neutralization of surface α-enolase by its specific antibody, crystal-protein binding assay, crystal face-specific binding determination, chemico-protein interactions analysis, and competitive binding assay using Mg^2+^ and divalent cation chelator.

## Results

**Figure 1:**
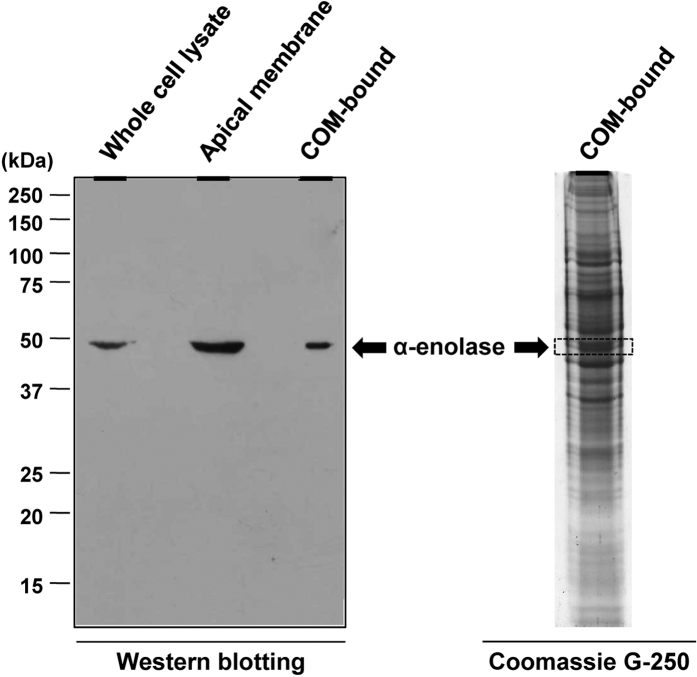
Western blot analysis of α-enolase. Proteins in whole cell lysate, apical membrane and COM crystal-bound fractions were resolved by 12% SDS-PAGE and subjected to Western blot analysis using rabbit polyclonal anti-α-enolase (Santa Cruz Biotechnology) as a primary antibody. Coomassie Brilliant Blue G-250-stained gel of the COM-bound fraction was also aligned with the immunoblot.

**Figure 2:**
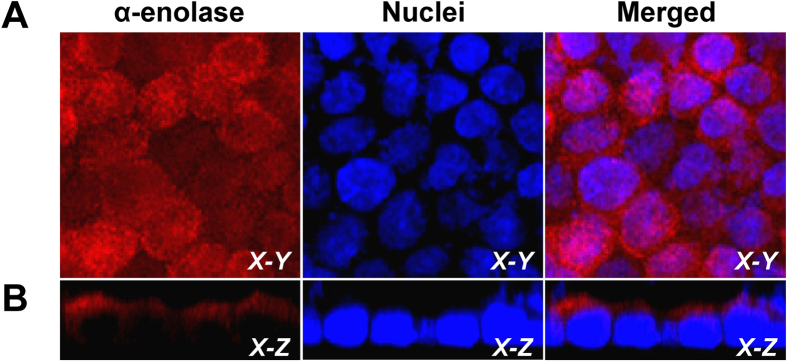
Confirmation of apical membrane localization of α-enolase on polarized MDCK cells. The polarized MDCK cell monolayer was fixed with 3.7% formaldehyde (without permeabilization) and then incubated with rabbit polyclonal anti-α-enolase antibody followed by incubation with Cy3-conjugated anti-rabbit IgG secondary antibody containing 0.1μg/ml Hoechst dye for nuclear staining. The confocal micrographs were obtained from horizontal (*X-Y*) sections at apical membranes **(A)** and also sagittal (*X-Z*) sections **(B)**. Original magnification power was 630X for all panels. Expression of α-enolase is shown in red, whereas nucleus is illustrated in blue.

Western blotting was performed to confirm the presence of α-enolase on apical membranes of MDCK renal tubular epithelial cells and also in COM crystal-bound fraction. Figure 1 shows that α-enolase was found in whole cell lysate, apical membrane and COM-bound fractions. Immunofluorescence staining and laser-scanning confocal microscopy were also performed to further validate apical surface localization of α-enolase in polarized MDCK cells. Polarized MDCK cells were fixed with 3.7% paraformaldehyde without any permeabilization step (to just demonstrate its surface localization, not the cytoplasmic expression) and incubated with rabbit polyclonal anti-α-enolase antibody. The confocal micrographs clearly illustrated apical surface localization of α-enolase (Fig. 2). These data strengthen the apical membrane localization of α-enolase in epithelial cells in addition to cytoplasm, which is its main localization.

**Figure 3:**
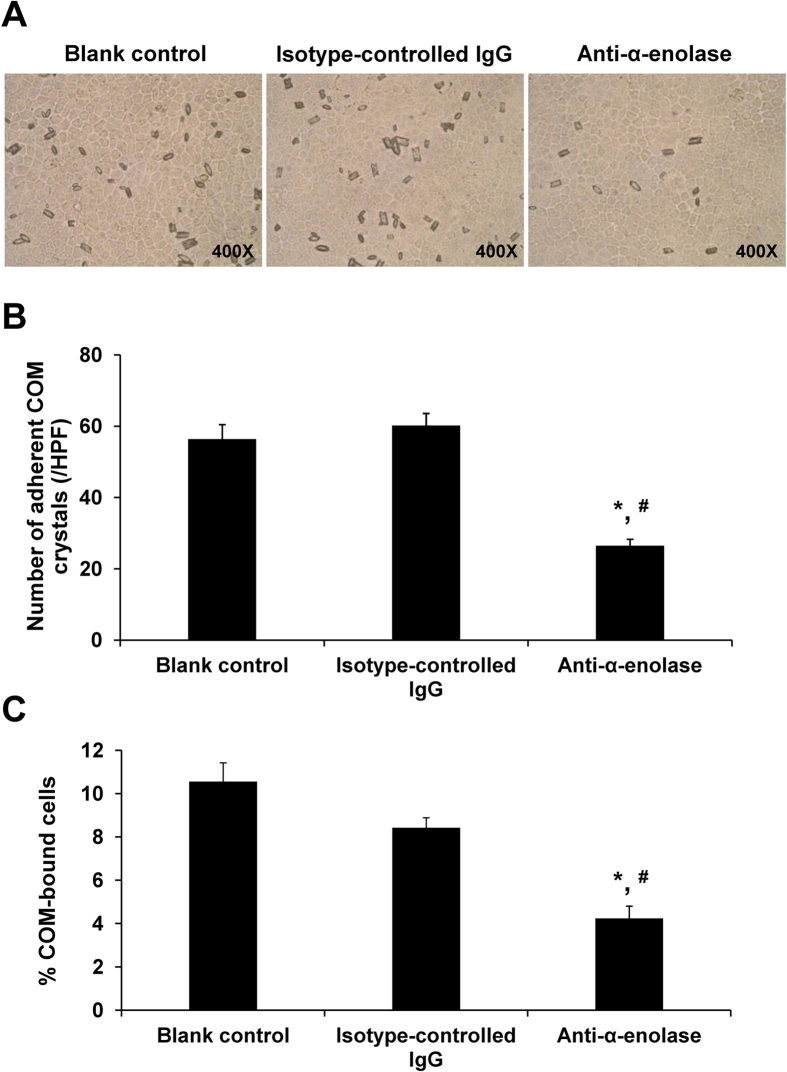
Cell-COM crystal adhesion assay and neutralization by a specific anti-α-enolase antibody. The confluent polarized cell monolayer was incubated with 0.2 μg/ml anti-α-enolase antibody or 0.2 μg/ml rabbit isotype-controlled IgG prior to cell-crystal adhesion assay (see details in “Materials and Methods”), whereas the cells without antibody pretreatment served as the blank control. **(A**) After removal of unbound crystals, the adherent crystals remained on the cell surface were imaged by a phase-contrast microscope. (**B**) The adherent crystals were counted from at least 15 random high power fields (HPFs). (**C**) Percentage of COM-bound cells was also determined. Original magnification power was 400X. Each bar represents mean ± SEM of the data obtained from 3 independent experiments. *p < 0.05 vs. blank control; #*p* < 0.05 vs. isotype-controlled IgG.

To further validate the role of surface α-enolase as a COM crystal receptor, its expression on cell surface was neutralized by incubating with rabbit polyclonal anti-α-enolase specific antibody prior to COM crystal administration in cell-COM crystal adhesion assay. After 1-h incubation with COM crystals, the unbound crystals were eliminated by extensive washes with PBS and the remaining crystals were observed under a phase-contrast microscope (Fig. 3A) and counted in at least 15 random high power fields (HPFs) (Fig. 3B). Neutralizing the surface α-enolase on MDCK cells significantly decreased the number of adherent COM crystals by approximately half of that observed in the blank control and isotype-controlled IgG groups (Fig. 3B). Similarly, the percentage of COM crystal-bound cells was also reduced by specific antibody neutralization by over a half as compared to the blank control and isotype-controlled IgG (Fig. 3C).

**Figure 4:**
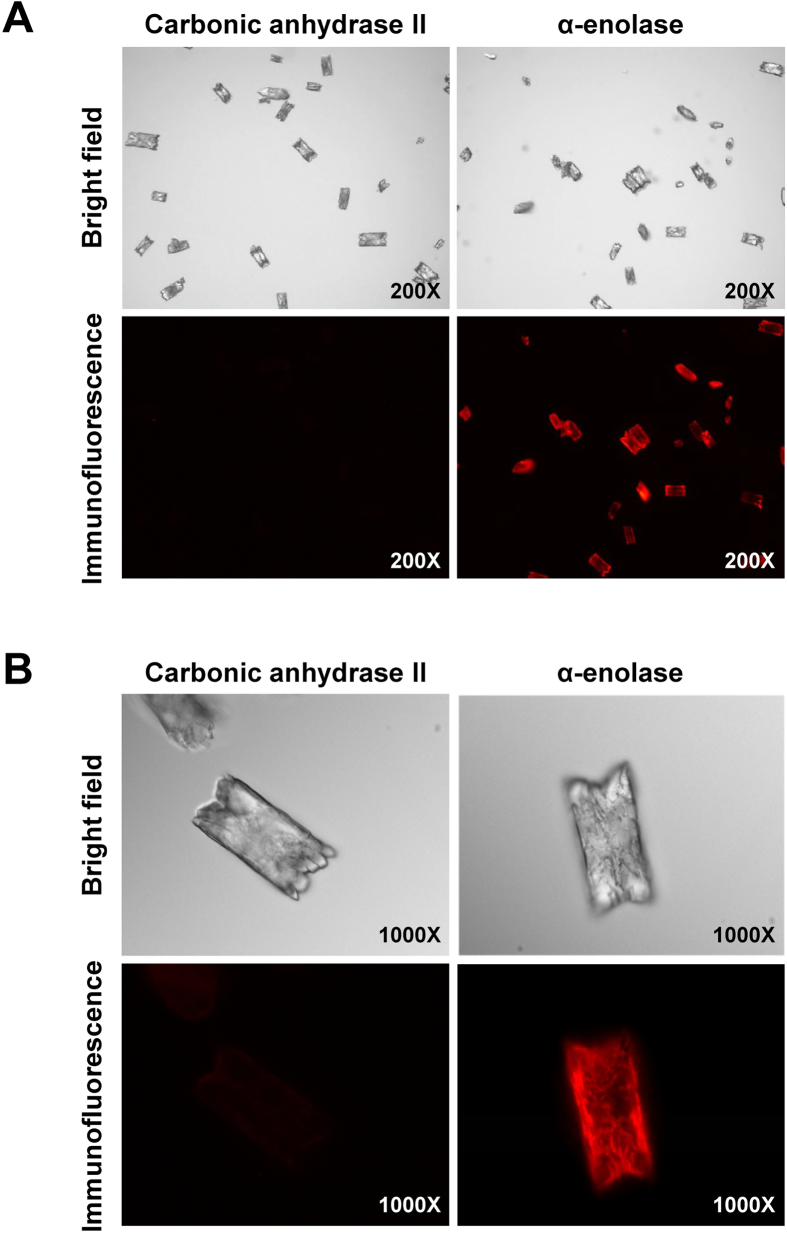
COM crystal-protein binding assay. Purified α-enolase or carbonic anhydrase II with an equal amount (5 μg) was directly incubated with COM crystals at 4 °C overnight. After washing, COM-bound protein was detected by immunofluorescence staining. **(A)** An overview of the immunofluorescence staining (original magnification power = 200X). **(B)** Zoom-in images of the immunofluorescence stained protein (shown in red in lower panels) comparing to the bright field (upper panels) (original magnification power = 1,000X).

We next addressed whether the binding of α-enolase to COM crystal specific or not. COM crystals were incubated with purified protein α-enolase and its binding to crystal surfaces was verified by immunofluorescence staining. The data obviously showed immunofluorescence staining of α-enolase (in red) on COM crystal surfaces, whereas carbonic anhydrase II, which was used as a negative control, showed no binding to COM crystal surfaces (Fig. 4). These findings indicated that α-enolase specifically bound to COM crystals and their interactions might be determined by particular domain on the α-enolase molecule that could capture Ca^2+^ on the COM crystal surfaces.

**Figure 5:**
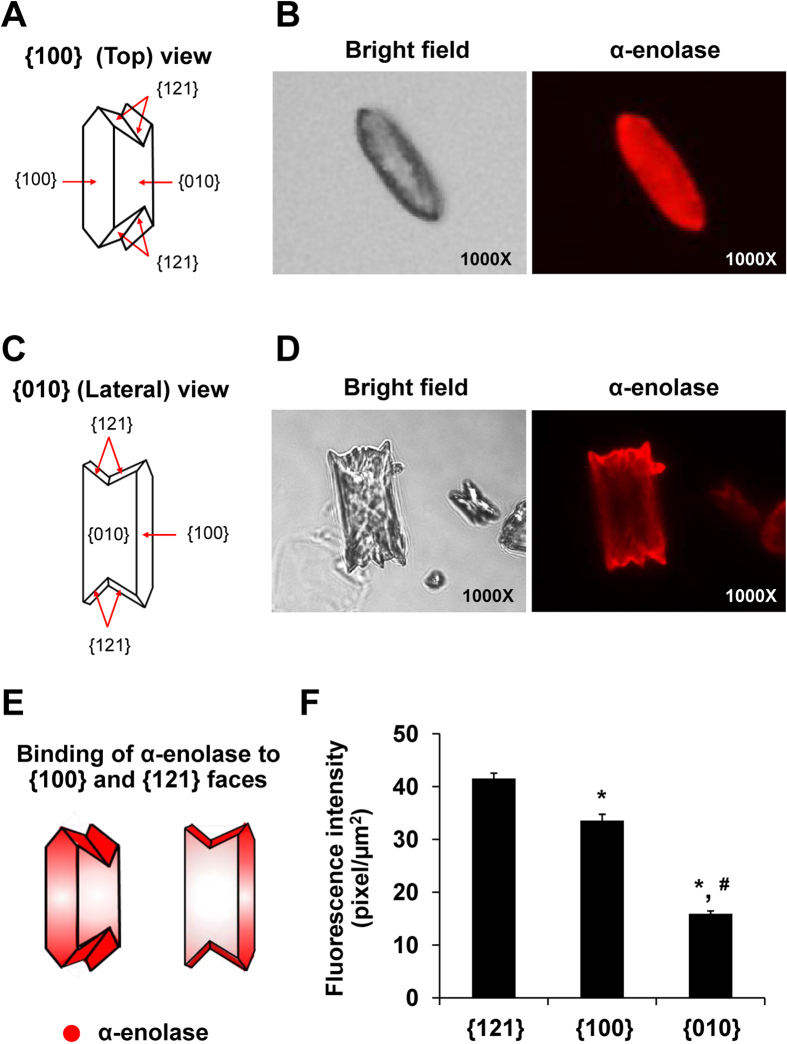
Analysis of α-enolase binding on different faces of COM crystal. Purified α-enolase (5 μg) was directly incubated with COM crystals at 4 °C overnight. After washing, α-enolase bound on each face of COM crystal was evaluated by immunofluorescence staining (shown in red) in top **(A,B)** and lateral **(C,D)** views. Quantitative analysis of immunofluorescence staining of α-enolase bound on each face of COM crystal was performed using ImageJ software from at least 100 individual COM crystals **(E,F)**. Original magnification power = 1,000X in all panels. Each bar represents mean ± SEM of the data obtained from 3 independent experiments.*p < 0.05 vs. {121} face; #*p* < 0.05 vs. {100} face.

COM crystals, which were generated in an inorganic solution, showed three common crystal faces, including {100}, {010} and {121} (Fig. 5A,C). Binding of purified α-enolase on these different crystal faces was then evaluated (Fig. 5B,D). Top and lateral views of immunofluorescence images showed the preferential binding of purified α-enolase on {121} as compared to {100} and {010} (Fig. 5E,F). These data indicated face-specific binding feature of α-enolase onto COM crystals.

**Figure 6:**
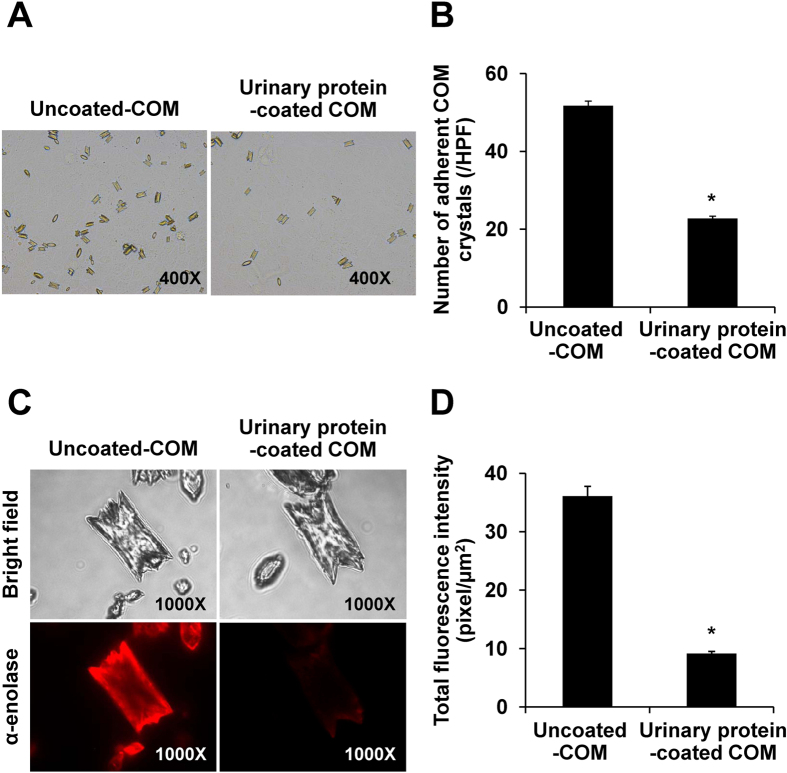
Effects of urinary proteins on cell-COM crystal adhesion and crystal-protein binding. (**A**,**B**) Cell-COM crystal adhesion assay (original magnification = 400X). The adherent crystals were counted from at least 15 random high power fields (HPFs). **(C,D)** Crystal-protein binding assay (original magnification = 1,000X). Quantitative analysis of immunofluorescence staining of α-enolase bound on COM crystals in each group was performed using ImageJ software from at least 100 individual COM crystals per group. Each bar represents mean ± SEM of the data obtained from 3 independent experiments. *p < 0.05 vs. uncoated-COM crystal.

Human urine normally composes of a lot of macromolecules, particular urinary proteins, which may have affect binding between COM crystals and α-enolase on renal tubular epithelial cells. We thus examined the effect of urinary proteins on COM crystal adhesion by coating COM crystal surface with urinary proteins prior to cell-COM crystal adhesion and crystal-protein binding assays. The data showed less binding capacity of the urinary proteins-coated crystals to adhere with MDCK cells as compared to the uncoated crystals (Fig. 6A,B). In addition, coating the crystals with urinary proteins dramatically reduced binding of the purified α-enolase onto COM crystal surfaces (Fig. 6C,D). These data clearly demonstrated the interference of urinary macromolecules to the binding of COM crystals and surface α-enolase on renal tubular epithelial cells.

**Figure 7:**
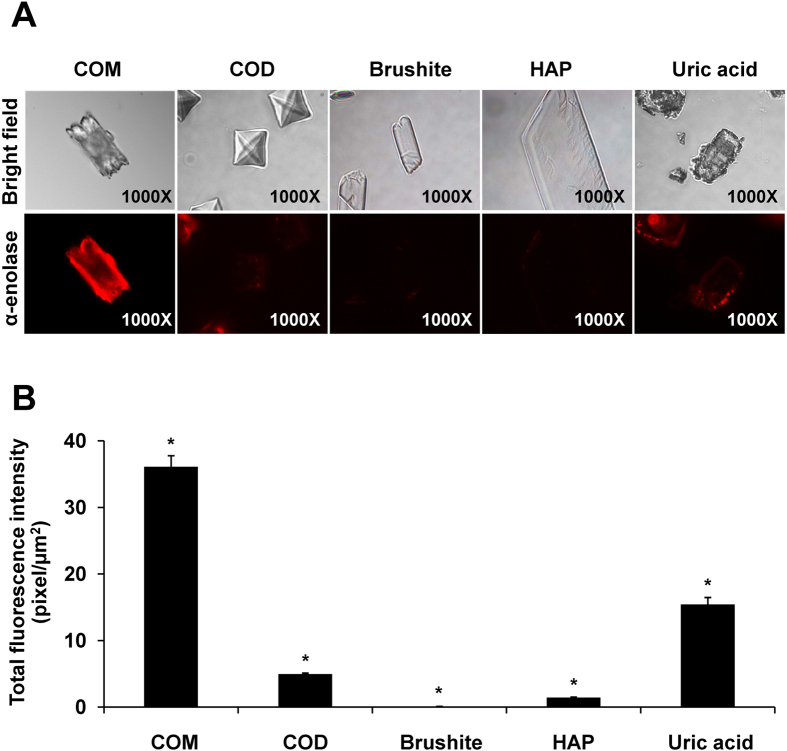
Binding of α-enolase on differential crystal types. Purified α-enolase (5 μg) was directly incubated with COM, COD, brushite, HAP or uric acid crystals using an equal amount of crystals (5 mg/ml artificial urine) at 4 °C overnight. **(A)** After washing, crystal-bound α-enolase was detected by immunofluorescence staining (shown in red) (original magnification power = 1,000X). **(B)** Quantitative analysis of immunofluorescence stained α-enolase bound on each crystal type was performed using ImageJ software from at least 100 individual crystals per group. Each bar represents mean ± SEM of the data obtained from 3 independent experiments. *p < 0.05 vs. other four crystal types.

Differential crystal types may have significant effects on α-enolase binding. We thus evaluated the specificity of binding of α-enolase on COM crystal surfaces by comparing with other crystal types, including calcium oxalate dihydrate (COD), brushite, hydoxyapatite (HAP) and uric acid crystals. Immunofluorescence data showed the most potent binding of α-enolase on COM crystals, whereas its binding to other crystal types was much weaker (Fig. 7). This data confirmed the specific binding of α-enolase onto COM, not other crystal types.

**Figure 8:**
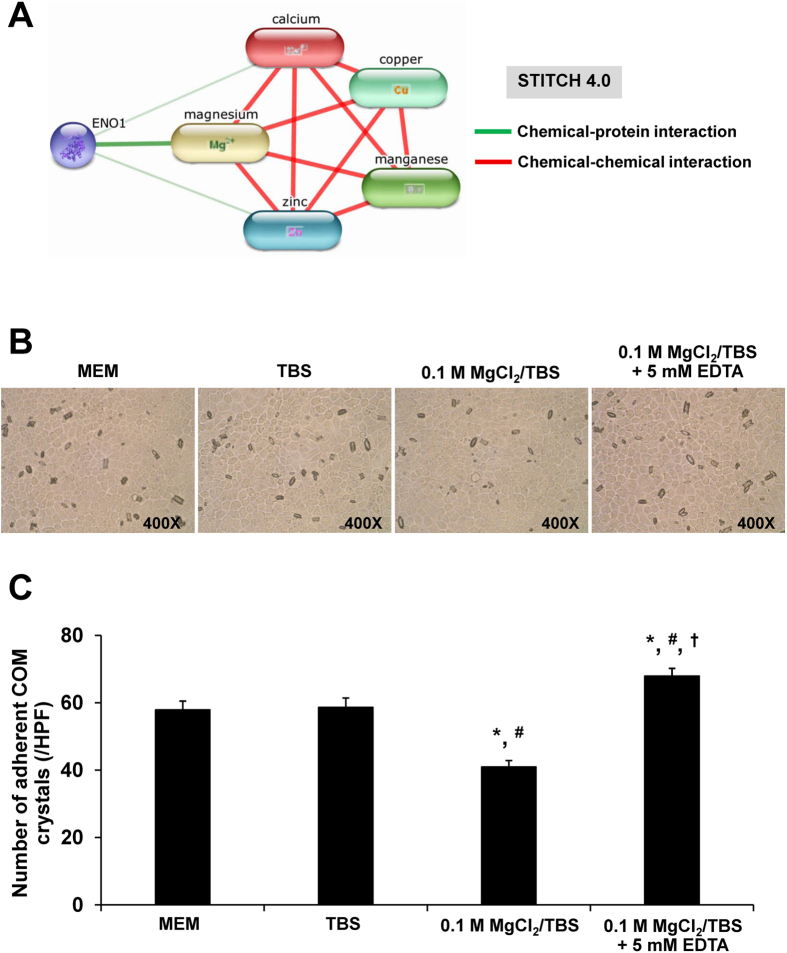
Chemico-protein interactions analysis and competitive binding assay using Mg^2 + ^. **(A)** Interactions between α-enolase (ENO1) and divalent cations commonly found in human urine (Ca^2+^, Mg^2+^, Mn^2+^, Zn^2+^ and Cu^2+^ ) were analyzed by using STITCH tool (version 4.0). **(B,C)** MDCK monolayers were pretreated with equal volume of MEM (blank control), Mg^2+^-free TBS (preservative or background control), or 0.1 M MgCl_2_ in Mg^2+^-free TBS for 30 min prior to cell-crystal adhesion assay. Moreover, the divalent cation-binding sites on α-enolase were recovered by further incubating the cells with 5 mM EDTA in Mg^2+^-free TBS for 15 min prior to cell-crystal adhesion assay. Original magnification power was 400X. Each bar represents mean ± SEM of the data obtained from 3 independent experiments. *p < 0.05 vs. MEM, #*p* < 0.05 vs. TBS; ^†^*p* < 0.05 0.1 M MgCl_2_/TBS.

A-enolase has three binding sites for divalent cations^15^,^16^. It is thus possible that various cations in the urine could competitively bind to apical surface α-enolase and affect COM crystal binding^6^,^16^. All the divalent cations commonly found in human urine via renal excretion including Ca^2+^, Mg^2+^, Mn^2+^, Zn^2+^ and Cu^2+^ 17, all of which play important roles in various biological processes, were subjected to chemico-protein interactions analysis to predict their interactions with α-enolase using STITCH tool version 4.0 ( http://stitch.embl.de/). Such interactions analysis revealed that α-enolase directly interacted with Ca^2+^, Zn^2+^ and, with a greater extent, Mg^2+^, which also interacted with all other cations (Fig. 8A). From this chemico-protein interactions network, the data suggested that Mg^2+^, which is a major abundant cation in the urine, is a potent α-enolase binder and thus could interfere with binding between α-enolase and COM crystal. We then examined whether blocking cation-binding sites of α-enolase on apical membranes of polarized renal tubular epithelial cells by Mg^2+^ could diminish cell-COM crystal adhesion or not. Moreover, we also addressed whether a divalent cation chelator could abolish or neutralize effect of Mg^2+^ competitive binding or not.

The cells were pretreated with equal volume of MEM (blank control), Mg^2+^-free TBS (preservative or background control), or 0.1 M MgCl_2_ in Mg^2+^-free TBS for 30 min prior to cell-COM crystal adhesion assay. The data showed significant reduction of the number of adherent crystals on the cells pretreated with Mg^2+^ as compared to the blank and background controls (Fig. 8B,C). Moreover, the data also demonstrated that EDTA could completely abolish the competitive effect of Mg^2+^ (Fig. 8B,C). These data strongly suggested that divalent cation-binding sites of α-enolase on apical surface of renal tubular epithelial cells play a critical role in mediating COM crystal adhesion.

## Discussion

Adhesion of COM crystals onto apical surface of renal tubular epithelial cells has been thought to be an early phase of stone nidus and kidney stone formation^18^,^19^,^20^. Our previous expression proteomics study identified α-enolase as a COM crystal-binding protein on apical membranes of renal tubular epithelial cells by quadrupole time-of-flight (Q-TOF) tandem mass spectrometry (MS/MS)^4^. Additional supportive evidence has demonstrated that expression level of α-enolase was increased and decreased after renal tubular cells were treated with stone aggravators^12^,^13^ and inhibitor^14^, respectively. However, its precise role as the potential COM crystal receptor remained unclear. The present study thus aimed to validate the role of α-enolase as the COM crystal receptor on apical surface of renal tubular epithelial cells.

Enolase (EC 4.2.1.11), also known as phosphopyruvate dehydratase, is a multifunctional metalloenzyme that catalyzes interconversion of 2-phospho-D-glycerate (PGA) and phosphoenolpyruvate (PEP) in the glycolytic pathway^6^. This catalytic reaction requires divalent cation, particular magnesium (Mg^2+^), for function^15^,^16^. In mammals, there are three enolase isoenzymes, including α, β and γ, each of which is a homodimeric protein^21^. Α-enolase is generally found in a variety of tissues, including the kidney^22^. In renal tubules, α-enolase is expressed in almost all segments of the nephron, especially distal tubule and collecting duct^21^, which are the main tubular segments for COM stone nidus formation^23^. Our data demonstrating the apical membrane localization of α-enolase on MDCK renal tubular epithelial cells (Figs 1 and 2) were in concordance with findings of Bonilha *et al.*^24^ who used a proteomic approach to characterize proteome of the isolated retinal pigment epithelium microvilli from polarized retinal epithelial cells and found that α-enolase was one of the microvillar proteins.

Recently, α-enolase has been reported with a novel function as a potential plasminogen receptor to bind with plasminogen and activate plasminogen to produce plasmin and induce fibrinolysis^7^,^8^,^25^. In addition, surface α-enolase has been implicated in tissue invasion and metastasis of various cancer cells, including cervical, colon, adenocarcinoma, and breast cancers as a result of plasminogen activation^26^,^27^. Moreover, our previous study has also demonstrated that α-enolase is involved in plasminogen activation leading to COM crystal invasion into renal interstitium^28^. The findings from our present study demonstrating that α-enolase served as a COM crystal receptor to mediate cell-crystal adhesion strengthened its significant roles in kidney stone disease pathogenesis.

Approximately 2.5% of total dry weight of kidney stone composes of organic matrices, of which >50% are enriched with urinary proteins that can affect various steps of kidney stone formation^29^,^30^,^31^. The effects of urinary proteins on binding ability of COM crystals to α-enolase on renal tubular cells were examined (Fig. 6). Our findings were consistent with those reported in previous studies, which demonstrated that macromolecules present in human urine could block the adhesion of COM crystals to renal tubular cells by coating crystal surface to prevent crystal retention inside the kidney^29^,^32^,^33^.

The binding of proteins to COM crystals has been thought to be associated with atomic array orientation of chemical compositions on the crystal surfaces^34^,^35^,^36^. Face-specific binding analysis revealed that α-enolase preferentially bound to COM crystal surfaces with the following order: {121} > {100} > {010} (Fig. 5). Preferential binding of α-enolase to {121} and also {100} faces might be due to the high Ca^2+^ density on these two faces of COM crystals^37^. A-enolase is a symmetrical dimeric enzyme, in which each monomer has three binding sites to divalent cations^15^,^16^. Interestingly, previous studies have suggested that the presence of Mg^2+^ could affect the adhesion forces between COM crystals and renal tubular epithelial cells^38^,^39^,^40^,^41^,^42^. Moreover, the increase of urinary Mg^2+^ excretion could inhibit kidney stone formation by preventing crystallization of Ca^2+^ and oxalate (C_2_O_4_^2−^) ions in the urine^38^,^39^,^40^,^41^,^42^ and by inhibiting adhesion of COM crystals to renal tubular epithelial cells^39^,^43^. In this study, we thus used a high (supraphysiologic) concentration of Mg^2+^ (at 0.1 M) to neutralize the divalent cation-binding sites of surface α-enolase to decrease Ca^2+^ affinity prior to performing cell-COM crystal adhesion assay. The data showed that the number of the crystals adhered onto the cell surface was significantly reduced by Mg^2+^, whereas preincubation with EDTA, a divalent cation chelator, completely abolished Mg^2+^ effect (Fig. 8), indicating that COM and Mg^2+^ competitively bind to α-enolase via divalent cation-binding domain. This information implicates that manipulation of urinary compositions and/or intratubular microenvironment (i.e. by Mg^2+^ supplement/treatment) may be one of the strategies to prevent kidney stone formation or its recurrence.

Indeed, EDTA treatment following Mg^2+^ preincubation caused a slightly increase in number of COM crystals bound on the cell surface (Fig. 8C). This was not unexpected because EDTA could chelate not only exogenous Mg^2+^ that was added during preincubation but also existing Mg^2+^ and other divalent cations, which are normally bound onto the apical surface molecules/proteins, including α-enolase. This finding was consistent with previous studies reporting the usage of EDTA to remove divalent cations from cell membranes^44^,^45^. Thus, the free divalent cation-binding sites on α-enolase molecule were increased and more available to bind with COM crystals.

In summary, our present study investigated functional role of α-enolase on apical membranes of renal tubular epithelial cells in association with kidney stone pathogenesis by using Western blotting, immunofluorescence staining, laser-scanning confocal microscopy, cell-crystal adhesion assay, neutralization of surface α-enolase by its specific antibody, crystal-protein binding assay, crystal face-specific binding determination, chemico-protein interactions analysis, and competitive binding assay using Mg^2+^ and divalent cation chelator. All these investigations successfully confirmed the role of α-enolase as a COM crystal receptor to mediate COM crystal adhesion at apical membranes of renal tubular epithelial cells. It may also serve as a target for future stone prevention by blocking COM crystal-cell binding and stone nidus formation.

## Materials and Methods

### Cell cultivation and polarization

Madin-Darby Canine Kidney (MDCK) cell line originated from distal renal tubular segment^46^,^47^ was used in this study. MDCK cells were grown in Eagle’s minimum essential medium (MEM) (Gibco, Invitrogen Corporation; Grand Island, NY) supplemented with 10% fetal bovine serum (FBS), 1.2% penicillin G/streptomycin and 2 mM L-glutamine, and maintained in a humidified incubator at 37 °C under 5% CO_2_. To develop polarization, the cells at a density of 7.5 × 10^4^ cells/ml were seeded and grown on prewetted collagen-coated permeable polycarbonate membrane insert in Transwells (0.4 μm pore size) (Corstar; Cambridge, MA). The culture medium was refreshed every other day for four days or until they became fully polarized epithelial cells. The preparation of collagen-coated permeable polycarbonate membrane insert was done according to our previous study^4^.

### Preparation of COM crystals

COM crystals were prepared according to protocols established previously^48^,^49^. Briefly, 10 mM CaCl_2_·2H_2_O and 10 mM Na_2_C_2_O_4_ were mixed to make final concentrations of 5 mM and 0.5 mM, respectively, in Tris buffer containing 90 mM NaCl (pH 7.4). The mixture was incubated at room temperature (RT) (set at 25 °C) overnight and then centrifuged at 3,000 rpm for 5 min. COM crystal pellets were collected, resuspended in methanol and then centrifuged at 3,000 rpm for 5 min. Methanol was removed and COM crystals were air-dried and then de-contaminated by UV radiation for 30 min before use.

### Preparation of COD, brushite, hydoxyapatite (HAP), and uric acid crystals

COD crystals were prepared according to protocols established previously^48^,^50^. Briefly, 125 ml of 25.08 mM CaCl_2_·2H_2_O was homogeneously mixed with 250 ml of a solution containing 19.26 mM C_6_H_5_Na_3_O_7_·2H_2_O, 23.1 mM MgSO_4_·7H_2_O and 127.4 mM KCl under continuous stirring. The pH was adjusted to 6.5 using HCl and the mixture was further incubated at RT for 15 min. Thereafter, 125 ml of 6.4 mM Na_2_C_2_O_4_ was added into the solution and further incubated at RT for 15 min. The crystals were collected by centrifugation at 3,000 rpm for 5 min, washed with methanol, and air-dried.

Brushite crystals were prepared by adding 0.015 g CaCO_3_ into 50 ml of 0.4 M NaH_2_PO_4_·H_2_O under continuous stirring at RT until completely dissolved. The solution was then incubated at RT without stirring for 2 h to allow brushite crystal formation. The crystals were collected by centrifugation at 3,000 rpm for 5 min, washed with deionized water, and air-dried.

HAP crystals were prepared by adding 400 μl of 5 M NaOH into 150 ml of 0.17 M CaCl_2_ under continuous stirring at RT. Thereafter, 50 ml of 0.3 M KH_2_PO_4_ was added into the mixture and further incubated at RT for 5 h. The crystals were collected by centrifugation at 3,000 rpm for 5 min, washed with deionized water, and air-dried.

Uric acid crystals were prepared by dissolving uric acid powder (>99% purity, Sigma; St. Louis, MO) in hot (75 °C) deionized water (at a concentration of 0.15 mg/ml). The oversaturated uric acid solution was cooled down at RT and further incubated at RT overnight. The crystals were collected by centrifugation at 3,000 rpm for 5 min, washed with deionized water, and air-dried.

All the harvested crystal types were de-contaminated by UV radiation for 30 min before use.

### Isolation of apical membranes and extraction of apical membrane proteins

Apical membranes of the polarized MDCK cells were isolated by a peeling method^51^. Briefly, after the cells were maintained in Transwells for four days, the culture medium was removed and the polarized cells were rinsed twice with ice-cold membrane preserving buffer (1 mM MgCl_2_ and 0.1 mM CaCl_2_ in PBS). Thereafter, Whatman filter paper (0.18-mm-thick) (Whatman International Ltd.; Maidstone, UK) pre-wetted with deionized water was placed onto the polarized cell monolayer. After a 5-min incubation period, the filter paper was peeled out and the apical membranes retained at the filter paper surface were harvested by rehydration in deionized water and gentle scrapping. The apical membrane-enriched fraction was then lyophilized. Dried apical membrane was solubilized in 1X Laemmli’s buffer and then dialyzed against deionized water at 4 °C overnight with three changes. After lyophilization, apical membrane protein powder was stored at −80 °C until used.

### Binding of apical membrane proteins to COM crystals and separation of COM crystal-binding proteins

Apical membrane protein powder was resuspended into 1 ml protein-free artificial urine (containing 5 mM CaCl_2_, 200 mM urea, 4 mM creatinine, 5 mM Na_3_C_6_H_5_O_7_·2H_2_O, 54 mM NaCl, 30 mM KCl, 15 mM NH_4_Cl, 2 mM MgSO_4_·7H_2_O, and 9 mM Na_2_SO_4_; pH = 6.2 and osmolality = 446 mOsm/kg)^52^. Thereafter, 5 mg COM crystals were added and apical membrane proteins were allowed to interact with COM crystals in the artificial urine on a continuous rotator at 4 °C overnight. The crystal-protein complexes were then collected by a centrifugation at 3,000 rpm for 5 min at 4 °C and the unbound proteins were discarded. Thereafter, the crystal-protein complexes were washed three times with PBS and other three times with 4 mM EDTA in PBS. After the final wash with PBS, COM crystal-bound proteins were eluted by 1X Laemmli’s buffer and separated in 12% SDS-PAGE gel. The resolved COM crystal-binding proteins were visualized by Coomassie Brilliant Blue G-250 stain.

### Western blot analysis

To confirm the presence of α-enolase on apical membranes and in COM crystal-bound fraction, an equal amount (20 μg) of proteins derived from whole cell lysate, purified apical membrane, and COM-bound fraction was loaded in each lane and resolved by 12% SDS-PAGE. The resolved proteins were then transferred onto a nitrocellulose membrane (Whatman; Dassel, Germany) using a semidry transfer apparatus (Bio-Rad; Milano, Italy) at 75 mA for 1 h. Non-specific bindings were blocked with 5% skim milk in PBS at RT for 1 h. The membrane was then incubated with rabbit polyclonal anti-α-enolase antibody (Santa Cruz Biotechnology; Santa Cruz, CA) (1:1,000 in 5% skim milk/PBS) at 4 °C overnight. Thereafter, the membrane was washed three times with PBS and further incubated with swine anti-rabbit IgG conjugated with horse-radish peroxidase (Dako, Gostrup, Denmark) (1:2,000 in 5% skim milk/PBS) at RT for 1 h. The immunoreactive band was then visualized by SuperSignal West Pico chemiluminescence substrate (Pierce Biotechnology, Inc.; Rockford, IL) and autoradiography.

### Immunofluorescence staining and laser-scanning confocal microscopy

To confirm the apical surface expression of α-enolase, polarized MDCK cells were rinsed with ice-cold membrane preserving buffer (1 mM MgCl_2_ and 0.1 mM CaCl_2_ in PBS) and then fixed with 3.7% paraformaldehyde in PBS at RT for 15 min (without permeabilization). After extensive washing with membrane preserving buffer, MDCK cells were incubated with rabbit polyclonal anti-α-enolase antibody (Santa Cruz Biotechnology) (1:50 in 1%BSA/PBS) at 37 °C for 1 h. The cells were then rinsed with PBS three times and then incubated with Cy3-conjugated goat anti-rabbit IgG antibody (Jackson ImmunoResearch Laboratories, Inc.; West Grove, PA) containing 0.1μg/ml Hoechst dye (DNA staining for nuclear localization) (Sigma; St. Louis, MO) at 37 °C for 1 h. Thereafter, the stained MDCK cells were washed with PBS and mounted with 50% glycerol/PBS for subsequent examination using a laser-scanning confocal microscope. 3-D planes of X-Y, X-Z and Y-Z scans were captured under ECLIPSE Ti-Clsi4 Laser Unit (Nikon; Tokyo, Japan) equipped with NIS-Elements D V.4.11 (Nikon).

### Cell-COM crystal adhesion assay and neutralization by a specific anti-α-enolase antibody

To determine whether surface α-enolase played role in cell-crystal adhesion, surface α-enolase expression was blocked by pretreating the cells with rabbit polyclonal anti-α-enolase antibody (Santa Cruz Biotechnology) prior to cell-crystal adhesion assay. Briefly, the confluent polarized cell monolayer was incubated with 1% BSA in membrane preserving buffer for 15 min to block non-specific bindings. Thereafter, the cells were washed with membrane preserving buffer three times and then incubated with 0.2 μg/ml anti-α-enolase antibody or 0.2 μg/ml rabbit isotype-controlled IgG at 37 °C for 30 min. After washing with membrane preserving buffer, COM crystals (100 μg crystal/ml medium) were added onto the cells and incubated at 37 °C for 1 h. The unbound crystals were eliminated by five washes with PBS. Finally, the remaining adherent COM crystals on the cell monolayer were counted in at least 15 random high power fields (HPF) under a phase-contrast microscope (Olympus CKX41, Olympus Co. Ltd.; Tokyo, Japan) and percentage of COM-bound cells was also determined.

### Crystal-protein binding assay followed by immunofluorescence staining

To further confirm that COM crystal-binding ability of α-enolase is specific, we directly incubated purified α-enolase with COM crystals and detected the presence of α-enolase on the COM crystal surface using immunofluorescence staining. Briefly, 5 μg purified α-enolase (Sino Biological Inc.; Beijing, China) was resuspended in 1 ml of protein-free artificial urine (as detailed above). COM crystals (5 mg) were added into the protein suspension and then incubated on the orbital rotator at 4 °C overnight. After washing with PBS, α-enolase-bound COM crystals were incubated with rabbit polyclonal anti-α-enolase antibody (Santa Cruz Biotechnology) at a dilution of 1:50 in 1% BSA/PBS at 37 °C for 1 h. After washing with PBS, chicken anti-rabbit IgG conjugated with Alexa 555 (Invitrogen-Molecular Probes; Burlington, ON, Canada) at a dilution of 1:2,000 in 1% BSA/PBS was added and then incubated at 37 °C for 1 h. The presence of purified α-enolase on the COM crystal surface was detected under ECLIPSE 80i fluorescence microscope (Nikon H600L, Nikon). An equal amount of purified carbonic anhydrase II (Sigma-Aldrich; Singapore) served as a negative control in parallel experiments using of rabbit polyclonal anti-carbonic anhydrase II (Chemicon International; Hampshire, UK) and chicken anti-rabbit IgG conjugated with Alexa 555 (Invitrogen-Molecular Probes) as primary and secondary antibodies, respectively. ImageJ software ( http://imagej.nih.gov/ij/) was used to quantify the fluorescence intensity of α-enolase on individual faces ({100}, {010} and {121}) of COM crystals from at least 100 individual crystals.

To compare the binding ability of α-enolase on five different crystal types, including COM, COD, brushite, HAP and uric acid crystals, the experiments were done using exactly the same protocol as of COM crystals (as described in details above) using equal amount of crystals (5 mg/ml artificial urine).

### Coating urinary proteins on COM crystal surface

To examine effects of urinary proteins on binding ability of COM crystals to renal tubular epithelial cells and to α-enolase, COM crystals were coated with human urinary proteins isolated from healthy volunteers prior to cell-COM crystal adhesion and crystal-protein binding assays, respectively. All the experiments involved human subjects and clinical samples were conducted according to the international guidelines, i.e. the Declaration of Helsinki, the Belmont Report, and ICH Good Clinical Practice, and have been approved by Siriraj Institutional Review Board (approval no. Si650/2015).

Random mid-stream void urine collected from healthy individuals who had no recent medication were centrifuged at 500 × g at RT for 5 min to remove cellular debris and particulate matter and then dialyzed against deionized water at 4 °C overnight with three changes. The dialyzed urine was concentrated by lyophilization and urinary proteins were then resuspended in deionized water. Protein concentration was measured by the Bradford method using Bio-Rad protein assay (Bio-Rad; Hercules, CA). Urinary proteins at a concentration of 5 μg/ml were incubated with COM crystals in protein-free artificial urine (100 μg crystals/ml) at 4 °C overnight. Thereafter, the urinary proteins-coated crystals were washed with PBS five times and then applied to cell-COM crystal adhesion and crystal-protein binding assays (as described above) to compare with the uncoated crystals.

### Chemico-protein interactions analysis

To further confirm the interaction between α-enolase and Ca^2+^ on the COM crystal surface and to search for potential interactions between α-enolase and other cations commonly found in the urine^17^, associations among α-enolase and Ca^2+^, Mg^2+^, Mn^2+^, Zn^2+^ and Cu^2+^ were subjected to chemico-protein interactions analysis using STITCH tool (version 4.0) ( http://stitch.embl.de/)^53^. This tool analyzed interactions between proteins and chemicals using both experimental and mining data retrieved from various databases^53^.

### Competitive binding assay using Mg^2+^

The interaction between α-enolase and Ca^2+^ was also validated by a competitive binding assay using Mg^2+^. After 4 days of cultivation, culture medium was removed and MDCK cells were rinsed twice with Mg^2+^-free Tris-buffered saline (TBS) (containing 50 mM Tris-HCl and 150 mM NaCl, pH 7.5). The cells were then incubated with 0.1 M MgCl_2_ in Mg^2+^-free TBS for 30 min to block the divalent cation-binding sites of α-enolase on the cell surface prior to cell-crystal adhesion assay. To further evaluate the role of divalent cation-binding sites on α-enolase in mediating COM crystal adhesion, we recovered the divalent cation-binding sites by further incubating the cells with 5 mM EDTA in Mg^2+^-free TBS for 15 min before performing cell-crystal adhesion assay. MEM and Mg^2+^-free TBS served as the blank control and preservative/background control, respectively.

### Statistical analysis

Comparisons of more than two groups were performed using ANOVA with Tukey’s post-hoc multiple tests. *P* values less than 0.05 were considered statistically significant. All data are reported as mean ± SEM.

## Additional Information

**Publisher's note**: Springer Nature remains neutral with regard to jurisdictional claims in published maps and institutional affiliations.

**How to cite this article**: Fong-ngern, K. and Thongboonkerd, V. Alpha-enolase on apical surface of renal tubular epithelial cells serves as a calcium oxalate crystal receptor. *Sci. Rep.* **6**, 36103; doi: 10.1038/srep36103 (2016).
